# Glucose Homeostasis and Pancreatic Islet Size Are Regulated by the Transcription Factors Elk-1 and Egr-1 and the Protein Phosphatase Calcineurin

**DOI:** 10.3390/ijms24010815

**Published:** 2023-01-03

**Authors:** Gerald Thiel, Oliver G. Rössler

**Affiliations:** Department of Medical Biochemistry and Molecular Biology, Saarland University, Building 44, 66421 Homburg, Germany

**Keywords:** Elk-1, Egr-1, calcineurin, pancreatic β-cells, islet size, glucose homeostasis

## Abstract

Pancreatic β-cells synthesize and secrete insulin. A key feature of diabetes mellitus is the loss of these cells. A decrease in the number of β-cells results in decreased biosynthesis of insulin. Increasing the number of β-cells should restore adequate insulin biosynthesis leading to adequate insulin secretion. Therefore, identifying proteins that regulate the number of β-cells is a high priority in diabetes research. In this review article, we summerize the results of three sophisticated transgenic mouse models showing that the transcription factors Elk-1 and Egr-1 and the Ca^2+^/calmodulin-regulated protein phosphatase calcineurin control the formation of sufficiently large pancreatic islets. Impairment of the biological activity of Egr-1 and Elk-1 in pancreatic β-cells leads to glucose intolerance and dysregulation of glucose homeostasis, the process that maintains glucose concentration in the blood within a narrow range. Transgenic mice expressing an activated calcineurin mutant also had smaller islets and showed hyperglycemia. Calcineurin induces dephosphorylation of Elk-1 which subsequently impairs Egr-1 biosynthesis and the biological functions of Elk-1 and Egr-1 to regulate islet size and glucose homeostasis.

## 1. Introduction

**Figure 1:**
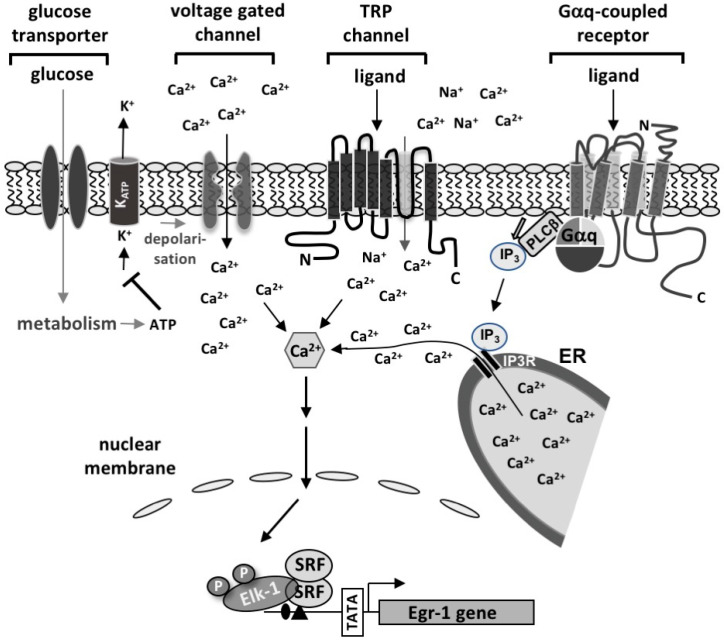
Cell surface receptors, channels, and transporters induce the phosphorylation of Elk-1 and the biosynthesis of Egr-1 in pancreatic β-cells. β-cells express numerous Ca^2+^ channels at the plasma membrane, i.e., transient receptor potential (TRP) channels and voltage-gated Ca^2+^ channels, which allow an influx of Ca^2+^ ions. Stimulation of Gαq-coupled receptors, such as M_3_ muscarinic acetylcholine receptors, increases the intracellular Ca^2+^ concentration via activation of phospholipase Cβ and the subsequent generation of inositol-1,4,5-trisphosphate (IP_3_). Glucose that enters the β-cells via glucose transporter is metabolized in the cells, resulting in increased ATP concentrations. ATP triggers a shut-down of nucleotide-responsive K^+^ channels. Subsequent depolarization of the plasma membrane leads to the activation of voltage-gated Ca^2+^ channels and an influx of Ca^2+^ ions. The increase in intracellular Ca^2+^ concentration is necessary for initiating stimulus-regulated insulin secretion. In addition, Ca^2+^ ions induce phosphorylation of Elk-1 and biosynthesis of Egr-1.

Pancreatic β-cells are specialized secretory cells that synthesize and secrete insulin, the key hormone for the control of energy metabolism, in a regulated manner. Cells must therefore be in close communication with their environment to sense extracellular signals that control insulin biosynthesis and secretion. β-cells express numerous receptors, transporters, and ion channels that link extracellular signals to intracellular responses (Figure 1). The concentration of glucose in the blood is the most important signal for triggering insulin secretion. To enter cells, the hydrophilic glucose molecules require specialized transporters.

Human β-cells express the glucose transporter GLUT1, whereas mice primarily express GLUT2 transporters in their β-cells. These transporters enable the influx of glucose into the β-cells. Glucose is metabolized in the cells via glycolysis, the TCA cycle, and the respiratory chain, leading to the biosynthesis of ATP. ATP shuts down nucleotide-responsive K^+^ channels (K_ATP_), thus preventing an efflux of K^+^ ions. As a result, the plasma membrane depolarizes, voltage-gated Ca^2+^ channels open, allowing an influx of Ca^2+^ ions into the cells. The increase of the intracellular Ca^2+^ concentration triggers the fusion of insulin-containing secretory granules with the plasma membrane that leads to the exocytosis of insulin. In particular, the Ca^2+^ channel Ca_v_1.2 has been shown to be of major importance for glucose-induced insulin release [1]. Voltage-gated Ca^2+^ channels can also be activated following an Na^+^-influx through transient receptor potential (TRP) TRPM3 channels [2]. Stimulation of G protein-coupled receptors additionally affects intracellular signaling cascades, for example by increasing intracellular Ca^2+^ concentration (Figure 1). In particular, Gαq-coupled receptors, such as M_3_ muscarinic acetylcholine receptors, play an important role in the regulation of insulin exocytosis and glucose homeostasis, i.e., the process that maintains blood glucose concentrations within a range between 4 mM and 8 mM [3].

## 2. Stimulation of Pancreatic β-Cells Induces the Expression of Egr-1

**Figure 2:**
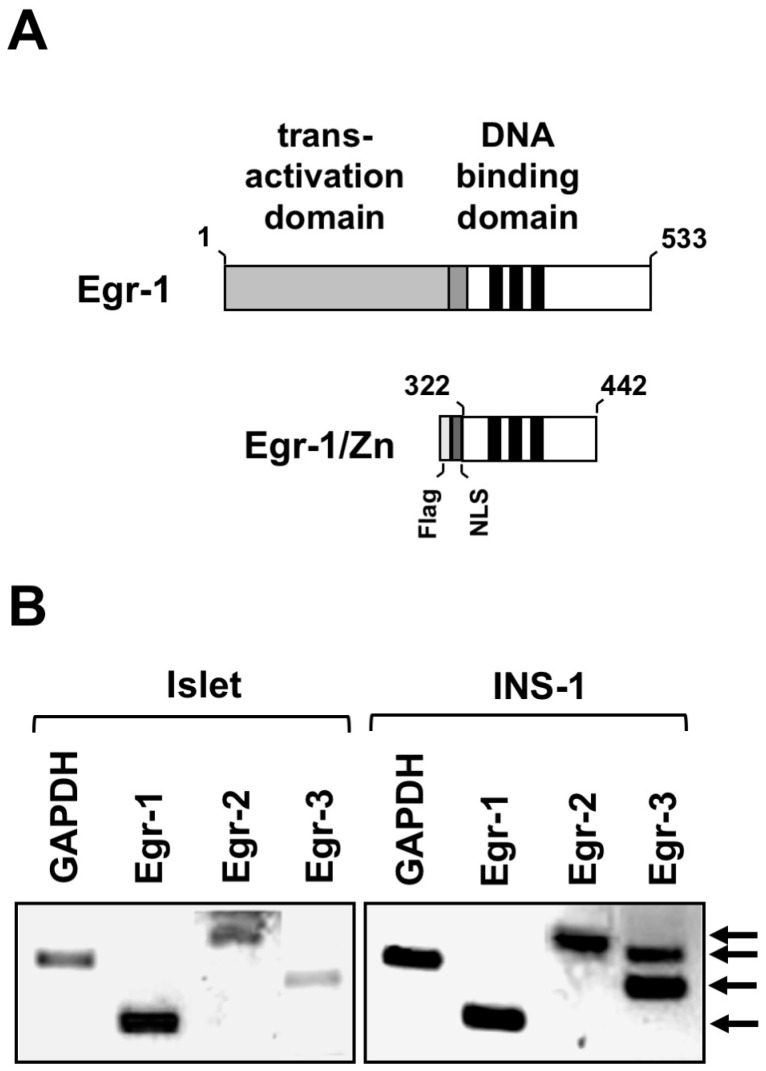
Domain structure of Egr-1 and Egr-1/Zn and expression of Egr proteins in pancreatic β-cells and insulinoma cells. (**A**) Domain structure of Egr-1 and Egr-1/Zn. The DNA binding domain of Egr-1 encompasses three zinc finger motifs. The activation domain is located N-terminal. Binding sites for NAB1 and NAB2, two negative cofactors, are adjacent to the DNA binding site. Egr-1/Zn is a truncated version of Egr-1, encompassing only the DNA binding domain. (**B**) Expression of the Egr proteins (Egr-1-3) in pancreatic islets and INS-1 insulinoma cells. Expression was detected by RT-PCR. As a control, GAPDH mRNA expression is depicted. Reproduced with modifications from ref. [10] with permission from Oxford University Press.

Higher glucose concentrations in the blood stimulate β-cells and trigger insulin biosynthesis and secretion. Glucose also stimulates the biosynthesis and activation of early growth response (Egr)-1, a transcription factor (Figure 2A) [4,5,6]. Egr-1 expression is also induced after stimulation of voltage-gated Ca^2+^ channels [6,7] or TRPM3 channels [7]. Moreover, stimulation of Gαq-coupled receptors that regulate insulin release and glucose homeostasis induces the expression of Egr-1 [8]. Additionally, Egr-1 expression is upregulated in insulinoma cells after stimulation of the glucagon-like peptide-1 (GLP-1) receptor with the GLP-1 agonist exendin-4 [9]. Figure 2B shows that Egr-1-3 proteins are expressed in pancreatic β-cells and INS-1 insulinoma cells [10]. Biosynthesis of Egr-2 is induced after stimulation of insulinoma cells with a synthetic agonist for voltage-gated Ca^2+^ channels [11], suggesting that glucose stimulation may also affect Egr-2 expression levels. It is not known whether glucose stimulation also induces Egr-3 biosynthesis in pancreatic β-cells. 

Egr-1 is not a β-cell-specific protein. Rather, Egr-1 is ubiquitously expressed, and many biological functions are known to be regulated by Egr-1, such as proliferation, reproduction, inflammation, and neuronal plasticity [12,13,14,15,16,17]. Egr-1 regulates insulin biosynthesis in pancreatic β-cells via activation of the homeobox transcription factor Pdx-1 [10,18,19]. The gene encoding Egr-1 contains several serum-response elements (SRE), and the major regulator of Egr-1 biosynthesis is the transcription factor Elk-1 (E twenty-six).

## 3. Stimulation of Pancreatic β-Cells Induces Phosphorylation and Activation of the Ternary Complex Factor Elk-1

**Figure 3:**
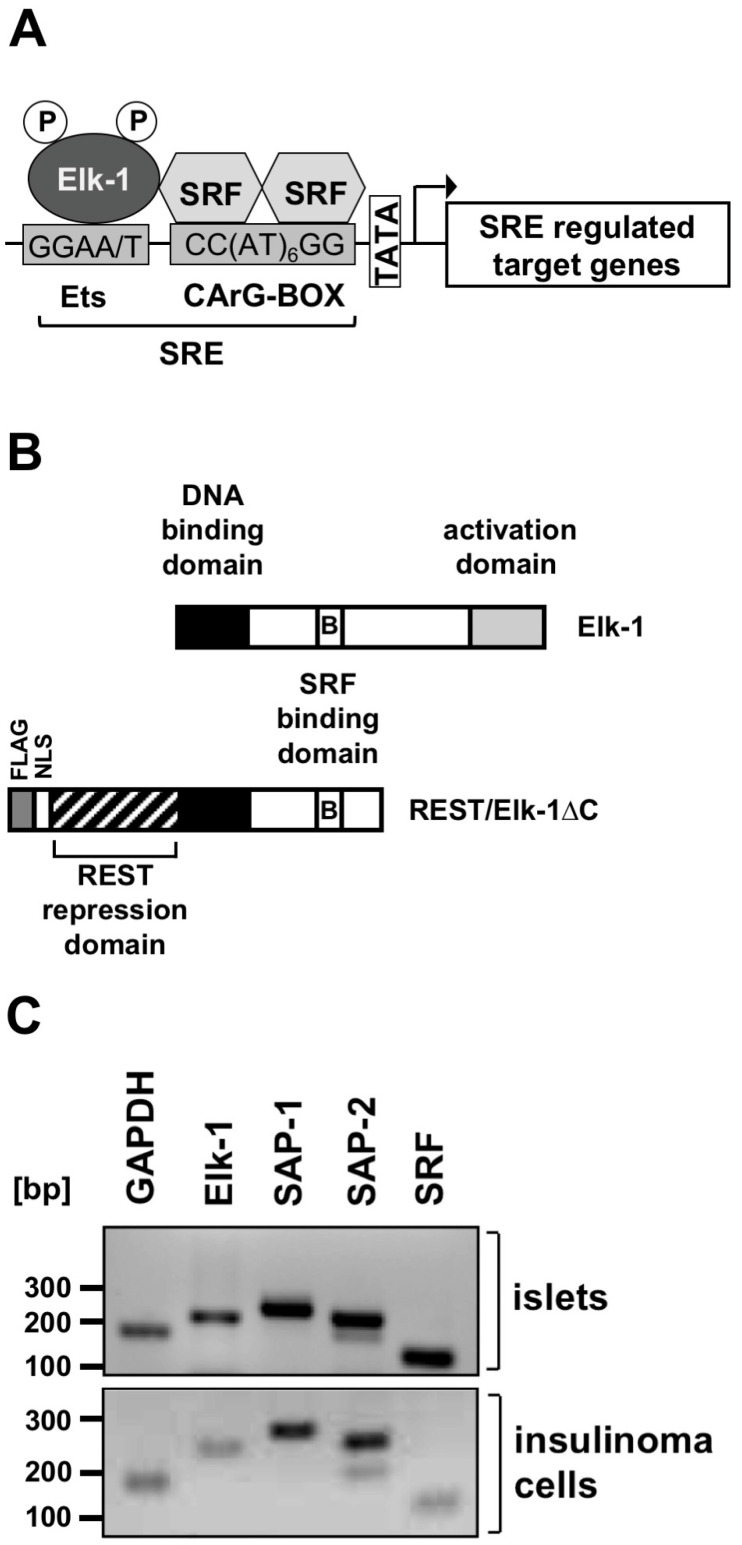
Domain structure of Elk-1 and REST/Elk-1∆C and expression of TCF proteins in pancreatic β-cells and insulinoma cells. (**A**) Schematic representation of the SRE that has interaction sites for Elk-1 and SRF. (**B**) Functional domains of Elk-1 and REST/Elk-1∆C. Elk-1 interacts with the DNA sequence GGAA/T via its DNA binding domain. Elk-1 binds to SRF via the B domain. The C-terminal activation domain contains major phosphorylation sites for the protein kinases JNK, ERK1/2, and p38. (**C**) RT-PCR analysis shows that Elk-1, the related TCF proteins SAP-1 and SAP-2, and the TCF interaction partner SRF are expressed in pancreatic islets and INS-1 insulinoma cells. Expression of GAPDH mRNA is also shown. Reproduced with modifications from ref. [26] with permission from Elsevier.

The transcription factor Elk-1 forms a ternary complex with a SRF (serum response factor) dimer that binds to the SRE (Figure 3A). Elk-1 is therefore also referred to as “ternary complex factor” (TCF). The SRE is a genetic element that links stimulation of the cells to Elk-1-mediated gene regulation. Elk-1 is phosphorylated by several protein kinases that act as signal transducer between the plasma membrane and the nucleus. Figure 3B shows the domain structure of Elk-1, which includes a DNA interaction domain, a binding domain for SRF (domain B), and an activation domain containing key phosphorylation sites. The transcriptional activity of the Elk-1/SRF ternary complex and subsequent transcription of SRE-regulated genes depends on the phosphorylation status of Elk-1. Elk-1 is therefore an important regulator of stimulus-transcription coupling [20]. Numerous signaling molecules induce the phosphorylation of Elk-1, i.e., ligands for receptor tyrosine kinases, G protein-coupled receptors, or cytokine receptors. The biological function of Elk-1 has been associated with the regulation of proliferation and development [16,21,22,23,24]. In pancreatic β-cells, phosphorylation of Elk-1 is induced by glucose [25]. Pancreatic β-cells express Elk-1 and the TCF proteins SAP-1 and SAP-2, as well as the TCF binding protein SRF (Figure 3C) [26].

## 4. Experimentally Overcoming Functional Redundancy: A Transgenic Strategy

Egr-1 and Elk-1 are proteins that belong to families of related proteins. The Egr proteins Egr-1-4 have homologous DNA binding sites and interact with similar GC-rich recognition sequences. Therefore, inactivation of one of the Egr encoding genes can be compensated by other Egr proteins [16,22,27]. The TCF family of proteins include Elk-1, SAP-1, and SAP-2, and the loss of a TCF protein can be compensated by related TCF proteins [22,28].

In similar scenarios, double or triple or quadruple knockout mice were generated to study the biological functions of a group of similar proteins. However, Egr-1 and Egr-4-deficient transgenic mice are sterile. 40% of Egr-3-deficient mice do not survive to postnatal day 21 [12,13,29]. Similarly, Elk-1 and SAP-1-deficient transgenic mice are infertile [22]. The generation of viable adult double or triple knockout mice targeting more than one Egr or TCF encoding genes is therefore difficult.

**Figure 4:**
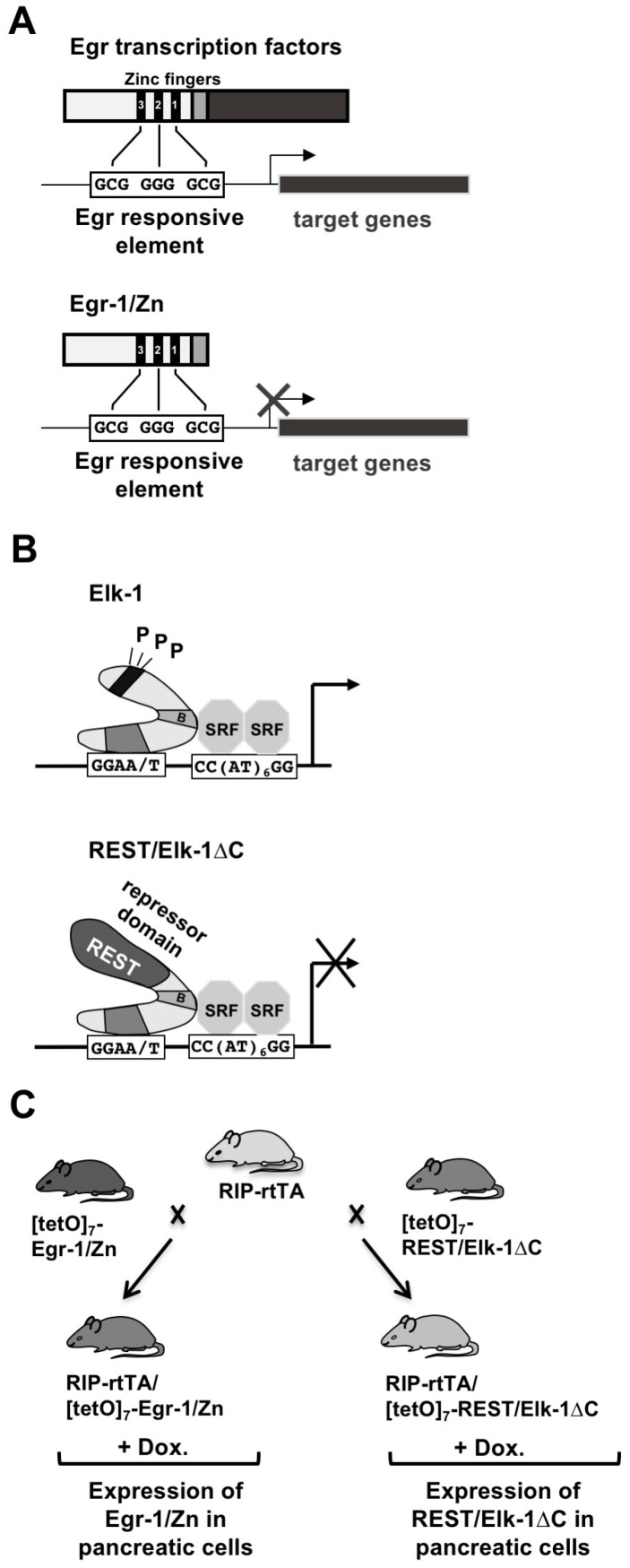
A transgenic strategy to overcome functional redundancy of protein families. (**A**) Egr-1/Zn functions as a dominant-negative for Egr proteins. Egr-1/Zn competes with Egr proteins for DNA binding. (**B**) REST/Elk-1∆C functions as a dominant-negative for TCF proteins. REST/Elk-1∆C competes with TCF proteins for DNA and SRF binding. In addition, the mutant induces chromatin compaction of SRE-regulated genes, due to the recruitment of histone deacetylases. (**C**) Crossing scheme to generate double transgenic mice expressing either Egr-1/Zn or REST/Elk-1∆C in pancreatic β-cells. Reproduced with modifications from refs. [10,26] with permission from Oxford University Press and Elsevier.

The problem of redundancy of biological functions of Egr or TCF proteins can be solved by different strategies. Tissue-specific inactivation of Egr or TCF encoding genes in pancreatic β-cells could solve the problem of lethality. Moreover, a dominant-negative approach does not change the expression levels of Egr and TCF proteins but rather affects the transcription of target genes regulated by Egr or TCF. The truncated Egr-1 mutant Egr-1/Zn, which contains only the zinc finger DNA interaction domain (Figure 2A), impairs DNA binding of all Egr proteins and thus transcription of Egr target genes (Figure 4A) [16]. Thus, it is not the expression of Egr proteins that is altered, but the transcription of Egr target genes. Accordingly, expression of Egr-1/Zn has been shown to interfere with the upregulation of Egr transcriptional activity following stimulation of voltage-gated Ca^2+^ channels in insulinoma cells [10].

REST/Elk-1∆C functions as a dominant-negative for Elk-1 (Figure 3B). The mutant contains the DNA and SRF interaction domains. However, the activation domain is removed. Therefore, the signaling cascade linking plasma membrane receptors to gene transcription is disrupted. This would require the phosphorylation of amino acid residues within the activation domain of Elk-1. In addition, REST/Elk-1∆C contains a transcriptional repression domain (derived from the transcriptional repressor REST) that recruits histone deacetylases to Elk-1 target genes. Histone deacetylases cause the compaction of chromatin. REST/Elk-1∆C competes with Elk-1 for binding to the SRE and the SRF, preventing the assembly of a functional ternary complex (Figure 4B). Expression of REST/Elk-1∆C does not affect TCF protein expression but rather impairs SRE-mediated transcription. REST/Elk-1∆C represses gene transcription regulated by all TCF proteins, because TCF proteins share high homology among themselves.

Egr-1/Zn and REST/Elk-1ΔC were expressed in β-cells of transgenic mice in a tissue-specific and inducible manner. The insulin promoter was used to express the mutant in pancreatic β-cells. The inducible Tet-on system was used to induce expression of the transgenes by adding the tetracycline derivative doxycycline to the drinking water. Three transgenic mouse lines were required for this strategy: [tetO]_7_Egr-1/Zn, [tetO]_7_REST/Elk-1ΔC, and RIP-rtTA. The first two lines express either Egr-1/Zn or REST/Elk-1ΔC, controlled by [tetO]_7,_ a tetracycline-responsive promoter. The RIP-rtTA mouse line expresses rtTA, the reverse tetracycline activator driven by the rat insulin II (RIP) gene promoter. Expression of rtTA is restricted exclusively to β-cells (Figure 4C). Thus, Egr-1/Zn and REST/Elk-1ΔC were expressed only in β-cells, controlled by the tetracycline derivative doxycycline (DOX).

## 5. Expression of Egr-1/Zn or REST/Elk-1∆C Leads to a Reduction in the Size of Pancreatic Islets

**Figure 5:**
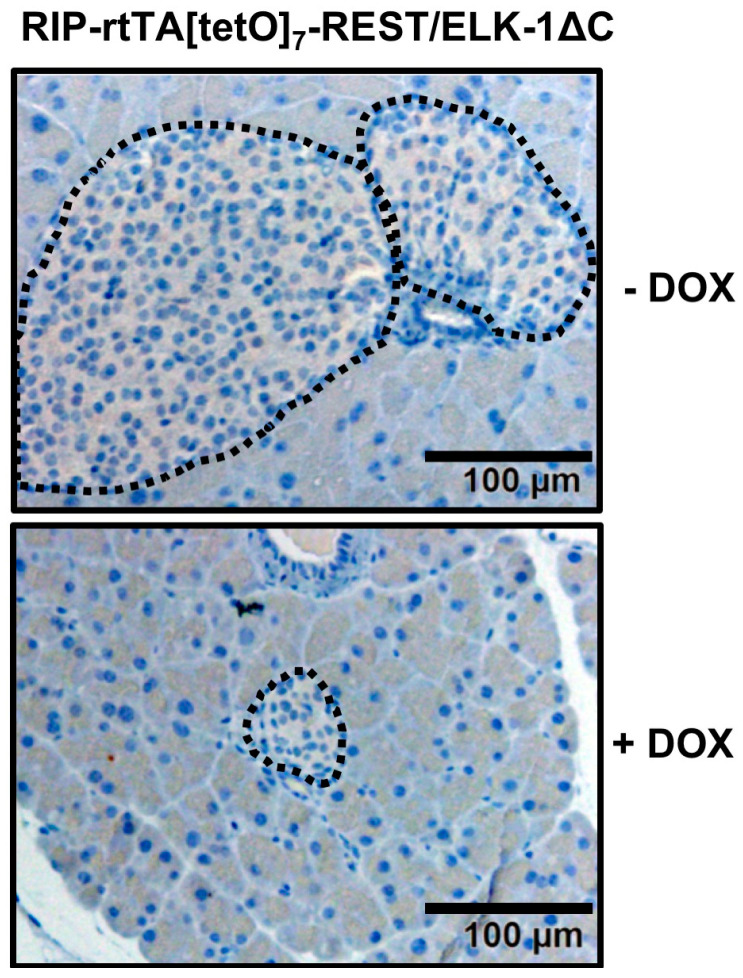
Formation of smaller islets in transgenic mice expressing REST/Elk-1ΔC in pancreatic β-cells. Sections of pancreata were stained with hematoxylin. Expression of REST/Elk-1∆C was induced by doxycycline (DOX) administered with drinking water. This was reproduced with modifications from [26] with permission from Elsevier.

Morphological analysis of transgenic mice expressing either the Egr-1/Zn or REST/Elk-1ΔC mice showed that the animals have significantly smaller pancreatic islets [10,26]. An example is shown in Figure 5. Quantification of islet size showed that β-cells-specific expression of Egr-1/Zn decreased islet size on the order of 20%, while expression of REST/Elk-1ΔC resulted in approximately 50% smaller islets compared to control mice. Further analysis showed that expression of the transgenes increased caspase-3 activity, suggesting that enhanced apoptosis was responsible for the generation of smaller islets. Interestingly, an increase in β-cell apoptosis has been proposed as a molecular mechanism responsible for the decrease in β-cell mass observed in patients with type 2 diabetes [30,31]. In contrast, physical exercise increases β-cell mass by stimulating β-cell proliferation and decreasing β-cell apoptosis [32]. Physical exercise stimulates extracellular signal-regulated protein kinase, an important activator of Egr-1 gene transcription, suggesting that Egr-1 may be involved in the execution of the benefits of physical training.

In addition, Egr-1 regulates the expression of the mitogen basic fibroblast growth factor (bFGF). Accordingly, transgenic mice expressing Egr-1/Zn in β-cells were found to have decreased bFGF expression [10]. Thus, increased apoptosis and decreased proliferation are responsible for the development of smaller islets.

## 6. Impaired Glucose Tolerance in Transgenic Mice Expressing Either Egr-1/Zn or REST/Elk-1ΔC in Pancreatic β-Cells

**Figure 6:**
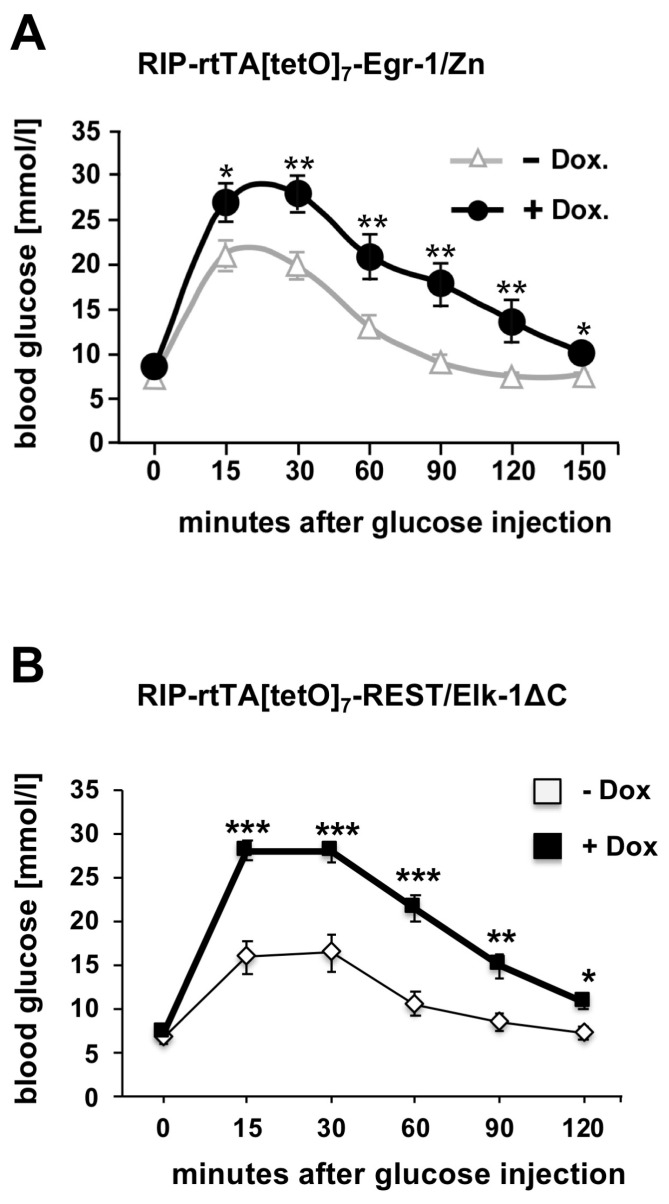
Glucose intolerance in transgenic mice expressing Egr-1/Zn or REST/Elk-1ΔC in pancreatic β-cells. Double transgenic RIP-rtTA/[tetO]_7_/Egr-1/Zn (**A**) or RIP-rtTA/[tetO]_7_REST/Elk-1ΔC mice (**B**) were used for glucose tolerance tests. Animals received doxycycline in the drinking water (+DOX) or not (−DOX). Statistics: +/− SEM, ✶, *p* < 0.05; ✶✶, *p* < 0.01; ✶✶✶, *p* < 0.001; *n* = 3). Reproduced with modifications from refs. [10,26] with permission from Elsevier and Oxford University Press.

Reduced numbers of β-cells have been identified as one of the determinants leading to the development of diabetes mellitus because there are not enough β-cells to synthesize and secrete sufficient insulin. A study of human pancreatic islets showed that three out of four type 2 diabetes mellitus patients had decreased β-cell mass and insulin expression [33]. Other studies examining patients with diabetes mellitus also found decreased β-cell mass [30,34]. It has been suggested that a 21% reduction in β-cell mass alone can lead to impaired glucose tolerance [35]. Transgenic mice expressing either Egr-1/Zn or REST/Elk-1ΔC showed decreased glucose tolerance (Figure 6) [10,26]. These observations support the association between a reduction in islet size and the development of glucose intolerance. In addition, Egr-1 regulates expression of Pdx-1 (pancreas duodenum homeobox-1) [10,18,19], a transcription factor that regulates insulin expression. Thus, impairment of Egr-regulated gene transcription directly affects insulin biosynthesis.

## 7. Expression of Activated Calcineurin A Leads to the Formation of Smaller Islets and Impaired Glucose Homeostasis

Transgenic mice expressing a constitutively active mutant of calcineurin A in β-cells exhibit hyperglycemia, decreased β-cell mass, decreased proliferation, and enhanced apoptosis [36], demonstrating a phenotype similar to that observed following expression of Egr-1/Zn or REST/Elk-1ΔC in β-cells. Calcineurin is a Ca^2+^/calmodulin-dependent protein phosphatase that is activated by increased intracellular Ca^2+^ ion concentrations. Elk-1 is an important substrate of calcineurin [37,38]. Since only phosphorylated Elk-1 is active, dephosphorylation catalyzed by calcineurin leads to its inactivation and impairment of Elk-1-mediated transcription. Experimentally, it was shown that expression of activated calcineurin A decreases the transcriptional activity of Elk-1 [39]. Since Elk-1 is a major inducer of Egr-1 biosynthesis, dephosphorylation of Elk-1 by calcineurin impairs Egr-1 expression in β-cells [7], cardiac muscles [40] and neurons [41,42]. Calcineurin thus negatively regulates SRE-mediated transcription and in this way inhibits stimulus-induced biosynthesis and activation of Egr-1.

## 8. Conclusions

**Figure 7:**
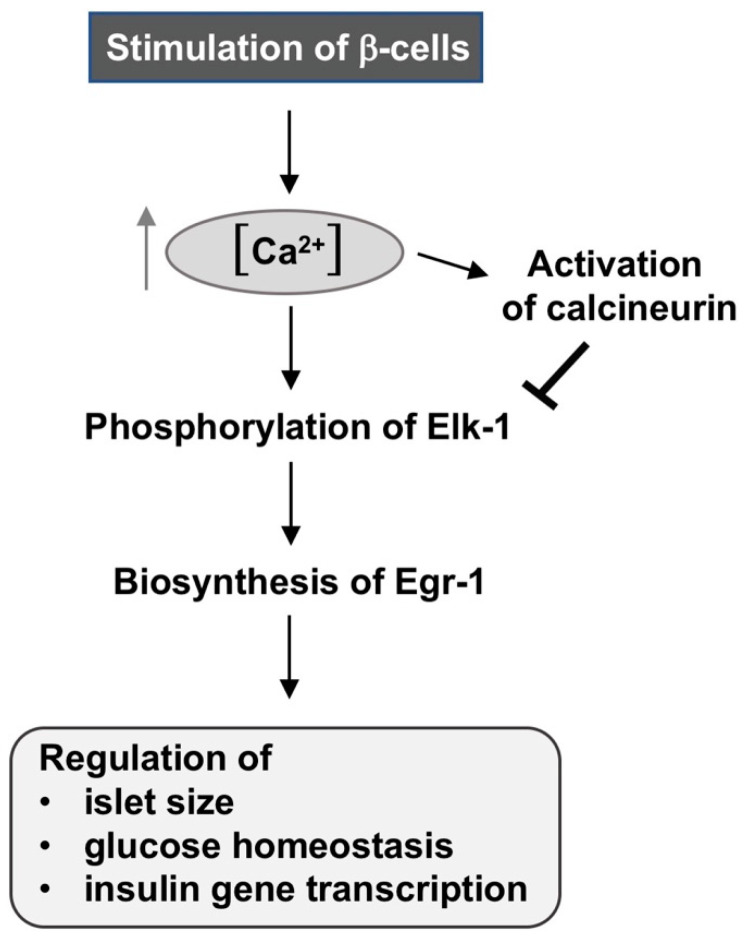
Sequential interplay of Elk-1, Egr-1, and calcineurin to regulate islet size and glucose homeostasis. Increase in intracellular Ca^2+^ ion concentration triggers phosphorylation of Elk-1 by Ca^2+^-responsive protein kinases. Elk-1 binds to different sites of the Egr-1 promoter and induces transcription of the Egr-1 gene. The newly synthesized Egr-1 protein regulates the formation of islets of sufficient size and thus glucose homeostasis. Calcineurin is activated by higher Ca^2+^ ion concentrations in cells and catalyzes dephosphorylation and inactivation of Elk-1.

In this review article, we focus on the number of β-cells as an important parameter to ensure that sufficient amounts of insulin are synthesized. A decreased number of β-cells has been identified as one of the determinants of the pathogenesis of diabetes mellitus [43]. An adequate number of β-cells is necessary for the biosynthesis of sufficient insulin. Therefore, the identification of regulatory proteins that determine the number of β-cells has a high priority for diabetes research. Experiments with transgenic mouse models showed that the stimulus-responsive transcription factors Elk-1 and Egr-1 are important for the formation of sufficiently large pancreatic islets. The mice exhibited glucose intolerance. Calcineurin negatively regulates the Elk-1—Egr-1 axis by dephosphorylating Elk-1 resulting in impaired Egr-1 biosynthesis (Figure 7).

The next step would be to analyze whether the identified proteins are druggable targets. We described in this article that basic research experiments have shown that the transcription factors Elk-1 and Egr-1 and the phosphatase calcineurin regulate the size of pancreatic islet in transgenic mice. Can these findings be transferred to humans and used for diabetes therapy? Pharmacological treatment of patients with diabetes mellitus currently focuses on stimulating insulin secretion. Interventions that leads to proliferation of β-cells in diabetic patients are difficult to design because an interference with the control mechanisms of β-cell growth is accompanied with an increased risk of tumor development. Egr-1 and Elk-1 transcription factors have been found to be responsible for the formation of islets of sufficient size, but transcription factors other than nuclear hormone receptors have been considered as undruggable targets.

Another problem is that Egr-1 and Elk-1 are expressed in different cell types and tissues, where they perform distinct tasks. Therefore, interventions have to be restricted to β-cells. Moreover, a gain-of-function intervention is required which is more difficult to archive than a loss-of-function intervention. In β-cells, a miRNA designated miR-375 has been identified as essential for maintaining β-cells mass [44]. This miRNA targets the 3’-untranslated region of the Elk-1 mRNA, resulting in translational repression of Elk-1. In smooth muscle cells, inhibition of miR-143 has been shown to induce Elk-1 expression and proliferation [45], suggesting that inhibition of miR-143 may be a strategy to increase Elk-1 activity in pancreatic β-cells. The transcriptional activity of Egr-1 is controlled by the co-repressor proteins NAB1 and NAB2, suggesting that inhibition of these proteins may prolong Egr-1 activity.

However, in order to use Elk-1 and Egr-1 as drug targets, many technical challenges need to be resolved. Significant progress has been made in synthetic biology and drug discovery [46]. Recently, an expression system was presented that shows how the pathway to manipulate gene transcription in therapeutics might look. The system is based on the expression of synthetic zinc finger transcription factors that allow inducible gene expression to be controlled by orthogonal small molecules [47].

## Data Availability

Not applicable.

## References

[1] Nitert M.D., Nagorny C.L.F., Wendt A., Eliasson L., Mulder H. (2008). Ca_v_1.2 rather than Ca_v_1.3 is coupled to glucose-stimulated insulin secretion in INS-1 832/13 cells. J. Mol. Endocrinol..

[2] Thiel G., Müller I., Rössler O.G. (2013). Signal transduction via TRPM3 channels in pancreatic β-cells. J. Mol. Endocrinol..

[3] Gautam D., Han S.-J., Hamdan F.F., Jeon J., Li B., Li J.H., Cui Y., Mears D., Lu H., Deng C. (2006). A critical role for β cell M_3_ muscarinic acetylcholine receptors in regulating insulin release and blood glucose homeostasis in vivo. Cell Metabol..

[4] Frödin M., Sekine N., Roche E., Filloux C., Prentki M., Wollheim C.B., Van Obberghen E.V. (1995). Glucose, other secretagogues, and nerve growth factor stimulate mitogen-activated protein kinase in the insulin-secreting beta cell line, INS-1. J. Biol. Chem..

[5] Josefsen K., Sorensen L.R., Buschard K., Birkenbach M. (1999). Glucose induces early growth response gene (Egr-1) expression in pancreatic beta cells. Diabetologia.

[6] Mayer S.I., Thiel G. (2009). Calcium influx into MIN6 insulinoma cells induces expression of Egr-1 involving extracellular signal-regulated protein kinase and the transcription factors Elk-1 and CREB. Eur. J. Cell Biol..

[7] Mayer S.I., Müller I., Mannebach S., Endo T., Thiel G. (2011). Signal transduction of pregnenolone sulfate in insulinoma cells. Activation of Egr-1 expression involving TRPM3, voltage-gated calcium channels, ERK, and ternary complex factors. J. Biol. Chem..

[8] Kaufmann K., Thiel G. (2002). Epidermal growth factor and thrombin induced proliferation of immortalized human keratinocytes is coupled to the synthesis of Egr-1, a zinc finger transcriptional regulator. J. Cell. Biochem..

[9] Kim M.-J., Kang J.-H., Chang S.-Y., Jang H.-J., Ryu G.R., Ko S.H., Jeong I.-K., Kim M.-S., Jo Y.-H. (2008). Exendin-4 induction of Egr-1 in INS-1 b-cells: Interaction of SRF, not YY1, with SRE site of rat Egr-1 promoter. J. Cell. Biochem..

[10] Müller I., Rössler O.G., Wittig C., Menger M.D., Thiel G. (2012). Critical role of Egr transcription factors in regulating insulin biosynthesis, blood glucose homeostasis and islet size. Endocrinology.

[11] Wang W., Walker J.R., Wang X., Tremblay M.S., Lee J.W., Wu X., Schultz P.G. (2009). Identification of small-molecule inducers of pancreatic β-cell expansion. Proc. Natl. Acad. Sci. USA.

[12] Lee S., Sadovsky Y., Swirnoff A.H., Polish J.A., Goda P., Gavrilina G., Milbrandt J. (1996). Luteinizing hormone deficiency and female infertility in mice lacking the transcription factor NGFI-A (egr-1). Science.

[13] Topilko P., Schneider-Maunoury S., Levi G., Trembleau A., Gourji D., Driancourt M.-A., Rao C.V., Charnay P. (1997). Multiple pituitary and ovarian defects in *Krox-24* (*NGFI-A, Egr-1*)-targeted mice. Mol. Endocrinol..

[14] Wei F., Xu Z.C., Qu Z., Milbrandt J., Zhuo M. (2000). Role of EGR1 in hippocampal synaptic enhancement induced by tetanic stimulation and amputation. J. Cell Biol..

[15] Yan S.-F., Fujita T., Lu J., Okada K., Zou Y.S., Mackman N., Pinsky D.J., Stern D.M. (2000). Egr-1, a master switch coordinating upregulation of divergent gene families underlying ischemic stress. Nat. Med..

[16] Mayer S.I., Rössler O.G., Endo T., Charnay P., Thiel G. (2009). Epidermal growth factor-induced proliferation of astrocytes requires Egr transcription factors. J. Cell Sci..

[17] Xie B., Wang C., Zheng Z., Song B., Ma C., Thiel G., Li M. (2011). Egr-1 transactivates Bim gene expression to promote neuronal apoptosis. J. Neurosci..

[18] Eto K., Kaur V., Thomas M.K. (2006). Regulation of insulin gene transcription by the immediate early response gene Egr-1. Endocrinology.

[19] Eto K., Kaur V., Thomas M.K. (2007). Regulation of pancreatic duodenum homeobox-1 expression by early growth response-1. J. Biol. Chem..

[20] Thiel G., Backes T.M., Guethlein L.A., Rössler O.G. (2021). Critical protein—Protein interactions determine the biological activity of Elk-1, a master regulator of stimulus-induced gene transcription. Molecules.

[21] Vickers E.R., Kasza A., Kurnaz I.A., Seifert A., Zeef L.A.H., O’Donnell A., Hayes A., Sharrocks A.D. (2004). Ternary complex factor-serum response factor complex-regulated gene activity is required for cellular proliferation and inhibition of apoptotic cell death. Mol. Cell. Biol..

[22] Costello P., Nicolas R., Willoughby J., Wasylyk B., Nordheim A., Treisman R. (2010). Ternary complex factors SAP-1 and Elk-1, but not Net, are functionally equivalent in thymocyte development. J. Immunol..

[23] Patki M., Chari V., Sivakumaran S., Gonit M., Trumbly R., Ratnam M. (2013). The ETS domain transcription factor ELK1 directs a critical component of growth signaling by the androgen receptor in prostate cancer cells. J. Biol. Chem..

[24] Gualdrini F., Esnault C., Horswell S., Stewart A., Matthews N., Treisman R. (2016). SRF Co-factors control the balance between cell proliferation and contractility. Mol. Cell.

[25] Bernal-Mizrachi E., Wen W., Srinivasan S., Klenk A., Cohen D., Permutt M.A. (2001). Activation of Elk-1, an Ets transcription factor, by glucose and EGF treatment of insulinoma cells. Am. J. Physiol. Endocrinol. Metabol..

[26] Lesch A., Backes T.M., Langfermann D.S., Rössler O.G., Laschke M.W., Thiel G. (2020). Ternary complex factor regulates pancreatic islet size and blood glucose homeostasis in transgenic mice. Pharmacol. Res..

[27] Tourtellotte W.G., Nagarajan R., Bartke A., Milbrandt J. (2000). 2000 Functional compensation by Egr4 in Egr1-dependent luteinizing hormone regulation and Leydig cell steroidogenesis. Mol. Cell. Biol..

[28] Cesari F., Brecht S., Vintersten K., Vuong L.G., Hofmann M., Klingel K., Schnorr J.J., Arsenian S., Schild H., Herdegen T. (2004). Mice deficient for the Ets transcription factor Elk-1 show normal immune responses and mildly impaired neuronal gene activation. Mol. Cell. Biol..

[29] Tourtellotte W.G., Milbrandt J. (1998). Sensory ataxia and muscle spindle agenesis in mice lacking the transcription factor Egr3. Nat. Genet..

[30] Butler A.E., Janson J., Bonner-Weir S., Ritzel R., Rizza R.A., Butler F.C. (2003). β-cell deficit and increased β-cell apoptosis in humans with type 2 diabetes. Diabetes.

[31] Marchetti P., Del Guerra S., Marselli L., Lupi R., Masini M., Pollera M., Bugliani M., Boggi U., Vistoli F., Mosca F. (2004). Pancreatic islets from type 2 diabetic patients have functional defects and increased apoptosis that are ameliorated by metformin. J. Clin. Endocrinol. Metab..

[32] Curran M., Drayson M.T., Andrews R.C., Zoppi C., Barlow J.P., Solomon T.P.J., Narendran P. (2020). The benefits of physical exercise for the health of the pancreatic β-cell: A review of the evidence. Exp. Physiol..

[33] Segerstolpe A., Palasantza A., Eliasson P., Andersson E.-M., Andréasson A.-C., Sun X., Picelli S., Sabirsh A., Clausen M., Bjursell M.K. (2016). Single-cell transcriptome profiling of human pancreatic islets in health and type 2 diabetes. Cell Metabol..

[34] Sakuraba H., Mizukami H., Yagihashi N., Wada R., Hanyu C., Yagihashi S. (2002). Reduced beta-cell mass and expression of oxidative stress-related DNA damage in the islet of Japanese type II diabetic patients. Diabetologia.

[35] Meier J.J., Breuer T.G., Bonadonna R.C., Tannapfel A., Uhl W., Schmidt W.E., Schrader H., Menge B.A. (2012). Pancreatic diabetes manifests when β cell area declines by approximately 65% in humans. Diabetologia.

[36] Bernal-Mizrachi E., Cras-Méneur C., Ye B.R., Johnson J.D., Permutt M.A. (2010). Transgenic overexpression of active calcineurin in β-cells results in decreased β-cell mass and hyperglycemia. PLoS ONE.

[37] Sugimoto T., Stewart S., Guan K.-L. (1997). The calcium/calmodulin-dependent protein phosphatase calcineurin is the major Elk-1 phosphatase. J. Biol. Chem..

[38] Tian J., Karin M. (1999). Stimulation of Elk1 transcriptional activity by mitogen-activated protein kinases is negatively regulated by protein phosphatase 2B (calcineurin). J. Biol. Chem..

[39] Langfermann D.S., Schmidt T., Rössler O.G., Thiel G. (2019). Calcineurin controls gene transcription following stimulation of a Gαq-coupled designer receptor. Exp. Cell Res..

[40] Schaeffer P.J., Wende A.R., Magee C.J., Neilson J.R., Leone T.C., Chen F., Kelly D.P. (2004). Calcineurin and calcium/calmodulin-dependent protein kinase activate distinct metabolic gene regulatory programs in cardiac muscle. J. Biol. Chem..

[41] Baumgärtel K., Genoux D., Welzl H., Tweedie-Cullen R.Y., Koshibu K., Livingstone-Zatchej M., Mamie C., Mansuy I.M. (2008). Control of the establishment of aversive memory by calcineurin and Zif268. Nature Neurosci..

[42] Lam B.Y.H., Zhang W., Enticknap N., Haggis E., Cader M.Z., Chawla S. (2009). Inverse regulation of plasticity-related immediate early genes by calcineurin in hippocampal neurons. J. Biol. Chem..

[43] Weir G.C., Gaglia J., Bonner-Weir Joslin S. (2020). Inadequate β-cell mass is essential for the pathogenesis of type 2 diabetes. Lancet Diabetes Endocrinol..

[44] Poy M.N., Hausser J., Trajkovski M., Braun M., Collins S., Rorsman P., Zavolan M., Stoffel M. (2009). *miR-375* maintains normal pancreatic α- and β-cell mass. Proc. Natl. Acad. Sci. USA.

[45] Cordes K.R., Sheehy N.T., White M.P., Berry E.C., Morton S.U., Muth A.N., Lee T.-H., Miano J.M., Ivey K.N., Srivastava D. (2009). miR-145 and miR-143 regulate smooth muscle cell fate and plasticity. Nature.

[46] Henley M.J., Koehler A.N. (2021). Advances in targeting ‘undruggable’ transcription factors with small molecules. Nature Rev. Drug Discov..

[47] Li H.-S., Israni D.V., Gagnon K.A., Gan K.A., Raymond M.H., Sander J.D., Roybal K.T., Joung J.K., Wong W.W., Khalil A.S. (2022). Multidimensional control of therapeutic human cell function with synthetic gene circuits. Science.

